# Locally superengineered cascade recognition–quantification zones in nanochannels for sensitive enantiomer identification†

**DOI:** 10.1039/d2sc03198a

**Published:** 2022-08-08

**Authors:** Junli Guo, Huijie Xu, Junjian Zhao, Zhida Gao, Zeng-Qiang Wu, Yan-Yan Song

**Affiliations:** College of Sciences, Northeastern University Shenyang 110819 China yysong@mail.neu.edu.cn; School of Public Health, Nantong University Nantong 226019 China zqwu@ntu.edu.cn

## Abstract

As an intriguing and intrinsic feature of life, chirality is highly associated with many significant biological processes. Simultaneous recognition and quantification of enantiomers remains a major challenge. Here, a sensitive enantiomer identification device is developed on TiO_2_ nanochannels *via* the design of cascade recognition–quantification zones along the nanochannels. In this system, β-cyclodextrin (β-CD) is self-assembled on one side of the nanochannels for the selective recognition of enantiomers; CuMOFs are designed as the target-responsive partners on the other side of the nanochannels for the quantification of enantiomers that pass through the nanochannels. As a proof-of-principle of the cascade design, arginine (Arg) enantiomers are tested as the identification targets. The l-Arg molecules selectively bind in the recognition zone; d-Arg molecules pass through the recognition zone and then interact with the quantification zone *via* a specialized reduction reaction. As verified by nanofluidic simulations, because of the confinement effect of nanoscale channels combined with the condensation effect of porous structure, the *in situ* reaction in the quantification zone contributes to an unprecedented variation in transmembrane K^+^ flux, leading to an improved identification signal. This novel cascade-zone nanochannel membrane provides a smart strategy to design multifunctional nanofluidic devices.

## Abstract 2

A design of the cascade recognition–quantification zone is developed along TiO_2_ nanochannels. The asymmetric nanochannels exhibit a predominant sensitivity and selectivity for enantiomer discrimination.

## Graphical abstract

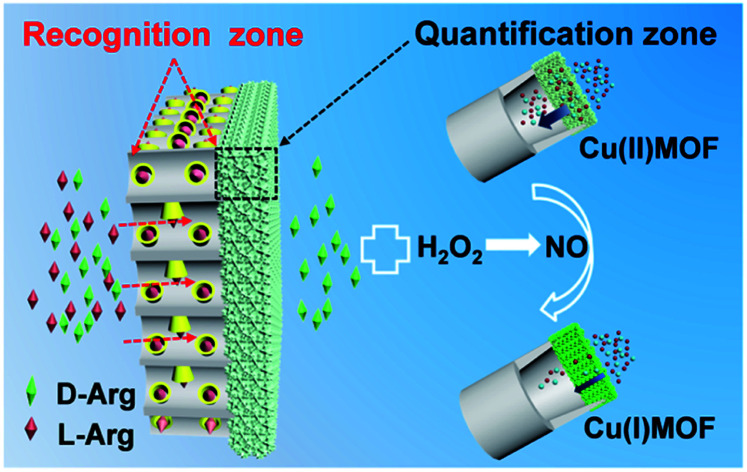

## Introduction

Chiral discrimination is a prominent feature of the living world. The body is amazingly chiral-selective, exhibiting different physiological responses to different enantiomers.^1,2^ Specifically, some molecules may produce the desired therapeutic activities, while their isomers may be inactive or produce unwanted effects. Amino acids are important bioactive substances. Studies on the enantiomeric recognition of amino acids can accelerate the understanding of chiral recognition in biological systems, thus promoting the development of designed molecular devices in biochemical and pharmaceutical fields.^3^ Although various strategies such as molecular imprinting,^4,5^ ligand exchange,^6,7^ and supramolecular interactions^8^ have been proposed for stereospecific molecular discrimination, enantioselective recognition of amino acids is still challenging because of similar physicochemical properties of optical isomers.^9,10^

Porous metal–organic frameworks (MOFs) represent a new class of inorganic–organic supramolecular hybrid materials comprising ordered networks formed from organic electron-donor linkers and metal cations.^11^ Their tunable pore size and characteristic functionality that are similar to those of the active sites in proteins suggest that they may act as promising host matrices for molecular recognition.^12^ In addition, considering the inherent confinement effect within their pores, MOFs can serve as a preconcentrator to enhance host–guest interactions. Furthermore, the surface designability of MOFs enables the incorporation of appropriate specific interaction sites into a scaffold using strategic organic chemistry techniques.^13^ Specific and unique molecular recognition between porous MOFs and guest substrates is the design criteria for the target recognition applications.^14^ In this case, a combination of signal transduction pathways and accessible MOF porosity will impart them with the capability of transducing the host–guest behavior to detectable changes; thus, porous MOFs are postulated as promising candidates for sensing applications.^15^ In general, to improve the performance of porous MOFs in enantiomer detection, it not only requires the enhancement of the detection ability of recognition units *via* specific host–guest interactions, but also involves constructing a reliable and sensitive signal transduction way that can provide information about host–guest interactions.^16^

Inspired by biological ion channels,^17,18^ artificial ion nanochannels with asymmetric structures have been widely constructed to mimic biological channels and applied in energy conversion,^19^ biochemical sensing,^20,21^ and other fields.^22,23^

Asymmetric artificial nanochannels with tailorable size and surface functionality are useful for mimicking ionic transport in biological ion channels.^24^ The changes in ionic transport characteristics can be directly monitored from the current–voltage (*I*–*V*) curves. Specifically, varying the asymmetric structure has been demonstrated as an effective way to induce remarkable changes in *I*–*V* properties.^25,26^ Recently, heterogeneous artificial nanochannels, in which a composite nanochannel has two or more chemical compositions, have attracted much attention because of their multiple functions, novel features, and operational feasibilities.^27–31^ These features are highly attractive for the design of sensors with a similar goal: to develop large numbers of low-cost sensors with sensitive performance to enable extensive application. Depending on the functions of individual materials, heterogeneous channels are largely desirable as a promising network for combining the enantiomer-recognition device and chiral-quantification device in a system. The development of sensitive enantiomer sensors is still a great challenge because of the small difference in the affinities for ligands between the target enantiomers.

Free-standing TiO_2_ nanotube/nanochannel arrays provide a new platform as artificial solid-state nanochannels. Particularly, the intrinsic photocatalytic properties of TiO_2_ make it possible to achieve subregional modification with two or more compositions in TiO_2_ nanotubes/nanochannels, thus achieving asymmetric heterogeneous nanochannels. Here, we investigated designs inspired by biological ion channels to develop enantioselective recognition sensors based on a free-standing TiO_2_ nanochannel membrane (TiO_2_M). The asymmetric TiO_2_M contains two different function zones along the nanochannel-enantiomer recognition zone and quantification zone. Arginine (Arg), an important functional molecule for cell division, human brain chemistry, immune responses, blood vessel dilation, and neurotransmission^32^ was applied as the target enantiomer. On one side of the nanochannels, β-cyclodextrin (β-CD) modification was performed for enantiomer recognition,^33^ which allowed one Arg enantiomer to pass through. On the other side of the nanochannels, the limit of light penetration in TiO_2_ materials was utilized to trigger the growth of Cu nanoparticles (CuNPs),^34^ which subsequently react with organic ligand 4,4′,4′′-tricarboxytriphenylamine (H_3_TCA) to generate CuTCA. When Arg reaches the quantification zone, it reacts with H_2_O_2_ to generate reductive NO,^35,36^ which further induces Cu(ii)-nodes on Cu-MOFs to produce Cu(i).^37^ This charge variation reported here was confirmed by a series of experimental studies from the transmembrane currents as well as the screening of fluorescence recovery of H_3_TCA ligands. Such a cascade system provides a smart, sensitive, and reliable strategy to design multifunctional devices.

## Results and discussion

### Fabrication and characterization of asymmetric membranes

**Fig. 1:**
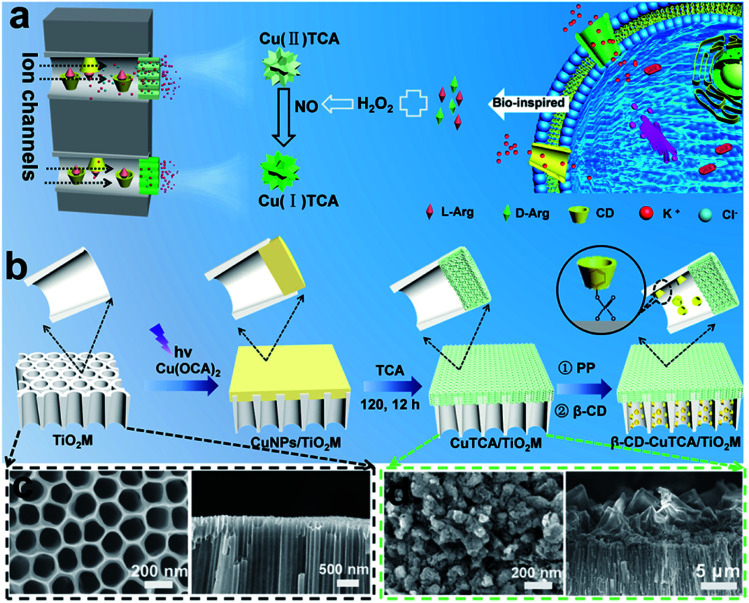
(a) Schematic illustration of β-CD–CuTCA/TiO_2_M and the principle of the recognition–quantification membrane. (b) Illustration of the preparation of β-CD–CuTCA/TiO_2_M from TiO_2_M. SEM images of the base entrance side and cross-section for (c) TiO_2_M and (d) CuTCA/TiO_2_M.

Inspired by biological ion channels, we designed asymmetric nanochannels composed of cascade recognition and quantification zones along the TiO_2_ nanochannels for the enantioselective detection of Arg enantiomers (Fig. 1a). The recognition zone was anchored with β-CD, a widely used host molecule for chiral recognition;^33^ the quantification zone was coated with CuTCA. The target enantiomers were transported from the recognition zone to the quantification zone. Owing to the larger specific affinity of β-CD in the recognition zone with one of the Arg enantiomers, the other enantiomer can more easily pass through the recognition zone and reach the quantification zone. In the quantification zone, these Arg molecules react with H_2_O_2_ to generate NO,^35,36^ which subsequently reduces the Cu(ii)-nodes on Cu-MOFs to Cu(i). The charge variation is asymmetrically located at the quantification zone of the nanochannels, which is expected to provide remarkable changes in the transmembrane ionic current.

**Fig. 2:**
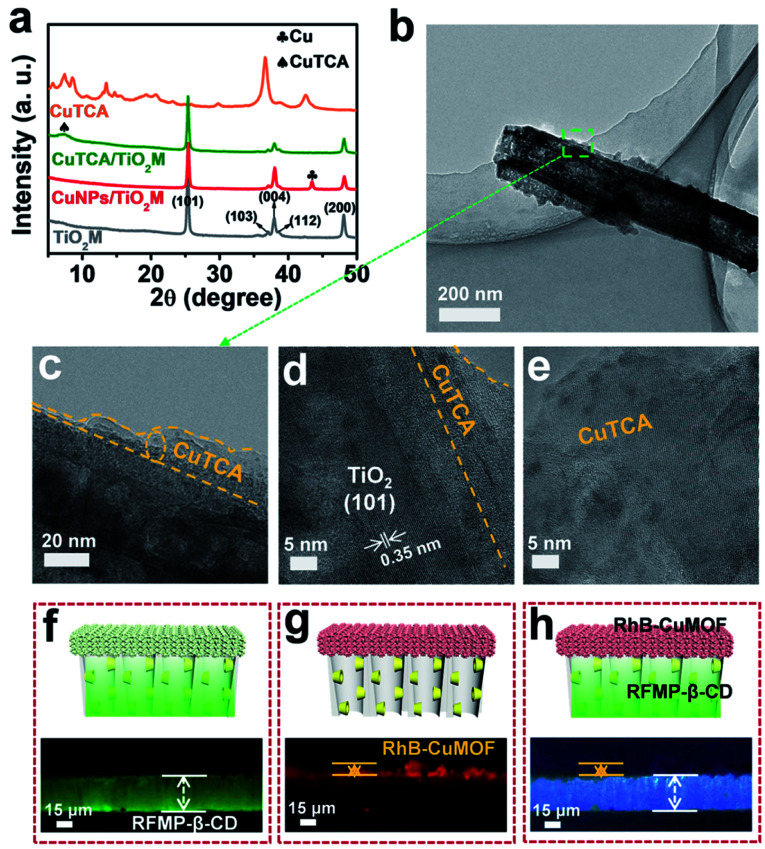
(a) XRD patterns of TiO_2_M, CuNPs/TiO_2_M, CuTCA/TiO_2_M, and CuTCA. (b–e) TEM images of β-CD–CuTCA/TiO_2_M. Fluorescence images of (f) the recognition zone (β-CD/TiO_2_M) labeled with RFMP (excitation wavelength 460–495 nm), (g) quantification zone (CuTCA/TiO_2_M) labeled with RhB (excitation wavelength 530–550 nm), and (h) whole asymmetric membrane (β-CD–CuTCA/TiO_2_M) under bright field.

Fig. 1b shows the procedure for preparing asymmetric nanochannels. TiO_2_M was fabricated by the electrochemical anodization of Ti foil in a lactic acid-containing glycerol/NH_4_F electrolyte (details are described in the Experimental section).^38,39^ The formed amorphous TiO_2_M was annealed at 450 °C in air for 2 h to remove the remaining organic electrolyte and meanwhile transform the amorphous TiO_2_ into the anatase phase, which has a better photocatalytic activity than the amorphous TiO_2_.^40,41^ The resulting pale-colour membrane implies that most of the contaminants inside the TiO_2_ nanotubes have been burned off (Fig. S1a and b†). As characterized by scanning electron microscopy (SEM), the as-formed TiO_2_M was composed of aligned nanochannels with a base entrance of 150 ± 20 nm in diameter (Fig. 1c) and a tip entrance of 40 ± 10 nm (Fig. S2a†). The membrane thickness was estimated to be ∼35 μm (Fig. S2b†), and the asymmetric structure can be further confirmed from the cross-section SEM images (Fig. S2c and d†). The selective decoration of CuNPs was achieved by combining the intrinsic photocatalytic activity of TiO_2_ nanochannels with a recently reported interfacial growth strategy.^42^ A bias (+1.0 V) was applied to drive the migration of Cu^2+^ ions from the tip entrance to the base entrance of TiO_2_M, and an LED (365 nm) was used to irradiate the base entrance side to trigger the photocatalytic reduction to form CuNPs. Because the light attenuation increases with the optical path length through the light absorber,^43^ CuNPs were mainly formed on the base entrance and the closed channel wall (Fig. S3†). To ensure the growth of CuNPs only on the base entrance side, experimental conditions such as the irradiation time (Fig. S4†) and Cu^2+^ concentration (Fig. S5a–d†) were optimized. Under the optimal conditions of 5.0 mM Cu^2+^ and 120 min LED irradiation, CuNPs were mainly found to be distributed on the base entrance and wall with a high density, and the depth of the CuNP layer was determined as ∼5.0 μm from the SEM image (Fig. S5e and f†). Using these CuNPs as the precursor of Cu^2+^, uniform and well-dispersed CuTCA nanocrystals were further fabricated on the base entrance side *via* a hydrothermal process in H_3_TCA.^37^ The resulting sample exhibits a green color (Fig. S1d†). As shown in Fig. 1e, the CuTCA nanocrystals appear on the tube wall close to the base entrance with a high density. No CuTCA was found on the tip entrance side (Fig. S6†), indicating successful asymmetric decoration. The transformation of CuNPs/TiO_2_M to CuTCA/TiO_2_M was confirmed from the X-ray diffraction (XRD) patterns and Fourier transform infrared (FTIR) spectra. In the XRD patterns, the presence of the characteristic peak of CuTCA at 7.5° and the disappearance of the characteristic peak of CuNPs at 43.5° also show the successful transformation of CuNPs to CuTCA (Fig. 2a). The FTIR bands at ∼778, 1172, 1273, and 1321 cm^−1^ are consistent with the absorption bands recorded for bulk CuTCA (Fig. S7†). In Fig. 2b, the transmission electron microscopy (TEM) images show the morphology of CuTCA/TiO_2_M, maintaining a typical nanochannel structure. In Fig. 2c–e, the high-resolution TEM (HRTEM) images show that the wall of the nanochannel is covered with a layer of small nanocrystals, and the characteristic spacing of 0.35 nm can be indexed to the (101) lattice plane of anatase TiO_2_ (JCPDS #21-1272).

To prepare the recognition zone, β-CD was used as the host molecule for Arg enantiomers and anchored onto TiO_2_ nanochannels from the tip side of the TiO_2_M using sodium phenyl phosphate (PP) as the connecting bridge. As shown in Fig. 1b, PP modification was first performed on the nanochannel wall *via* the well-known affinity interaction between phosphoric acid and Ti–OH groups.^44–46^ Then, β-CD was introduced onto the nanochannel wall through the host–guest interaction between β-CD molecules and phenyl on PP.^33^ The PP loading amount was found to depend on the pH of the PP solution (Fig. S8a†). The solution pH was optimized as pH 4 (Fig. S8b†), where the highest PP loading amount was achieved. The PP modification leads to the appearance of absorption bands at ∼1166 and ∼1740 cm^−1^ in the FTIR spectra (Fig. S9†), which are indexed to the P–O stretching and bending modes, respectively.^45^ X-ray photoelectron spectroscopy (XPS) analysis was carried out to confirm β-CD loading onto PP–TiO_2_M (Fig. S10†). Because of the presence of hydrocarbon groups on the β-CD molecule, the enhancement of C 1s signals (Fig. S10b†) and the decrease in the intensity of P 2p signals (Fig. S10c†) indicate the successful loading of β-CD onto PP–TiO_2_M.

To clearly show the spatial arrangement of β-CD and CuTCA on different sides of the nanochannels, β-CD/TiO_2_M and CuTCA/TiO_2_M zones were selectively dyed with two different fluorescent probes (Fig. 2f–h). Riboflavin sodium phosphate (RFMP), a green fluorescent probe, was labeled onto β-CD/TiO_2_M through the host–guest interaction between β-CD and thymine-like groups on RFMP.^33^ Rhodamine B (RhB, 0.56 nm × 1.18 nm × 1.59 nm), a typical red fluorescent probe, was encapsulated in the pores of CuTCA (the pore radius is about 1 nm).^37^ Upon excitation with a light of 460–495 nm, as shown in Fig. 2f, one side of the membrane shows brilliant green fluorescence, indicating the β-CD/TiO_2_M zone. Under an excitation wavelength of 530–550 nm, as shown in Fig. 2g, the other side of the membrane exhibits red fluorescence, indicating the CuTCA/TiO_2_M zone. Apparently, clearly divided regions with different colors are located on either side of the membrane (Fig. 2h). In addition, the energy-dispersive X-ray spectroscopy (EDS) images also verify that Cu and C elements mainly appear on one side of the as-proposed membrane, while the P element appears on the other side of the membrane (Fig. S11†). These results show the successful fabrication of the two functional zones on the different sides of TiO_2_M. The content of the Cu element in the resulting β-CD–CuTCA/TiO_2_M sample was further determined as 10.5 wt% by inductively coupled plasma-optical emission spectroscopy (ICP-OES) analysis.

### Enantioselective characterization of the membrane

**Fig. 3:**
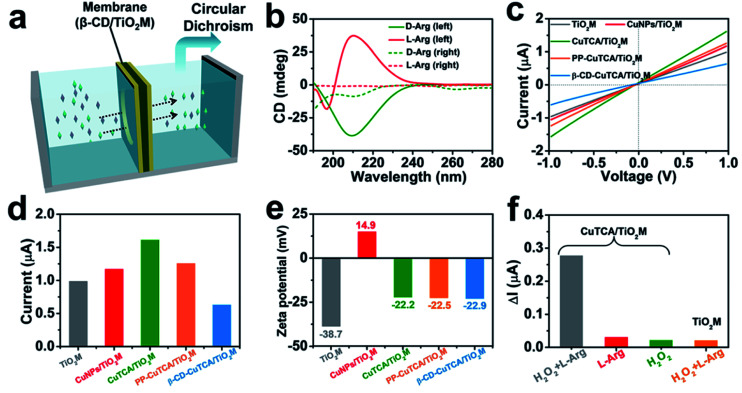
(a) Schematic illustration of Arg transport through the β-CD/TiO_2_M. (b) CD spectrum of 50 μM d/l-Arg (solid line) and d/l-Arg permeated solution after 24 h for β-CD/TiO_2_M (dashed line). (c) *I*–*V* curves for each step of modification. (d) The current values of each step of modification at +1.0 V. (e) Corresponding zeta potential of each step of modification. (f) Ionic current changes (Δ*I*) at +1.0 V of CuTCA/TiO_2_M in l-Arg with H_2_O_2_ (gray), l-Arg (red), and H_2_O_2_ (green), and Δ*I* at +1.0 V of TiO_2_M in l-Arg with H_2_O_2_ (yellow).

As a well-known enantioselective guest molecule, β-CD has a strong affinity with the enantiomer through the interaction of the polar amino and carboxyl groups of the target enantiomer with the hydroxyl groups of β-CD, thus forming hydrogen bonds at the mouth of the β-CD.^33,47^ To estimate the β-CD enantioselectivity-induced enantiomer transport difference in the as-proposed membrane, directional diffusion experiments were performed. A schematic setup of the diffusion experiment is shown in Fig. 3a. The β-CD/TiO_2_M (0.38 cm^2^ in area) was fixed in the middle of the two cells. The left cell was filled with 1.0 mL of 50 μM d-Arg or l-Arg solution, which served as the feed solution. The right cell only contained deionized water. Fig. 3b shows the circular dichroism (CD) spectra of the initial l/d-Arg in the left cell (solid lines) and the l/d-Arg in the right cell after a 24 h-diffusion experiment (dashed lines). Based on the absorption peak intensities, the crossed d-Arg is nearly 16 times the crossed l-Arg, verifying that d-Arg has much higher transport ability than l-Arg when passing through the recognition zone. This difference can be attributed to the different feasibility for hydrogen bonds because of the stereoselectivity (the steric hindrance at a chiral site), thus endowing β-CD-modified channels with the enantioselective ability.^47^

The *I*–*V* curves of the membranes were recorded using a home-made electrochemical cell (Fig. S12†). The as-prepared membrane was placed in the middle of two half cells, and two Ag/AgCl electrodes were inserted into the cell chamber containing 1.0 mM KCl. Fig. 3c shows the *I*–*V* curves of the membrane recorded at each step, and the corresponding ionic current changes at +1.0 V are shown in Fig. 3d. The transmembrane ionic current is enhanced after CuTCA growth, which can be attributed to the increased surface charge densities induced by the organic ligand H_3_TCA. Notably, after the subsequent PP and β-CD modification, the decrease in the ionic current can be observed in two steps. To gain a clear insight on the contribution of CuTCA, PP, and β-CD to the ionic current, zeta potentials of the membrane were measured at each step (Fig. 3e and S13†). TiO_2_M has a negatively charged surface with a zeta potential of −37.8 mV. CuNPs/TiO_2_M shows a positively charged surface (+14.9 mV), which can be attributed to the remaining Cu^2+^ adsorbed on the surface. Owing to the presence of plenty of –COOH groups on the H_3_TCA ligand, the growth of CuTCA results in a negatively charged surface (−22.2 mV). It should be noted that the zeta potential exhibits ignorable changes after modification with PP and β-CD. Therefore, a clear decrease in the ion flux of PP–CuTCA/TiO_2_M and β-CD–CuTCA/TiO_2_M could be related to the hydrophobicity and the steric hindrance of the phenyl group and β-CD.

The quantification ability of the CuTCA zone was evaluated from the *I*–*V* curves of CuTCA/TiO_2_M in a 1.0 mM KCl solution at room temperature using different concentrations of l-Arg as the target. The ionic currents were found to strongly decrease with the concentration of l-Arg in the presence of 1.0 mM H_2_O_2_ (Fig. S14a†), indicating that the amount of Cu(i) was dependent on the Arg concentration in the quantification zone. For comparison, the *I*–*V* curves of CuTCA/TiO_2_M were also recorded in the absence of H_2_O_2_ (Fig. S14b†) or l-Arg (Fig. S14c†), which showed ignorable changes in the ionic currents. This indicates that the reduction reaction was limited. In addition, negligible changes in the ionic currents were found for bare TiO_2_M in the presence of both l-Arg and H_2_O_2_ (Fig. S14d†), indicating that CuTCA was the key component for achieving a sensitive response in ion flux. To obtain satisfactory sensitivity in Arg quantification, the effect of solution pH and KCl concentration on transmembrane ionic currents was also optimized. For the Arg–H_2_O_2_ reaction, the generated NO is related to the pH of electrolyte (Fig. S15†) with a remarkable drop at pH 4. Additionally, larger ionic currents were recorded in higher concentrations of KCl solution (Fig. S16†). Therefore, the following *I*–*V* measurements were performed in 0.5 mM KCl (pH 4).

### Signal magnification in *I*–*V* curves for chiral recognition

**Fig. 4:**
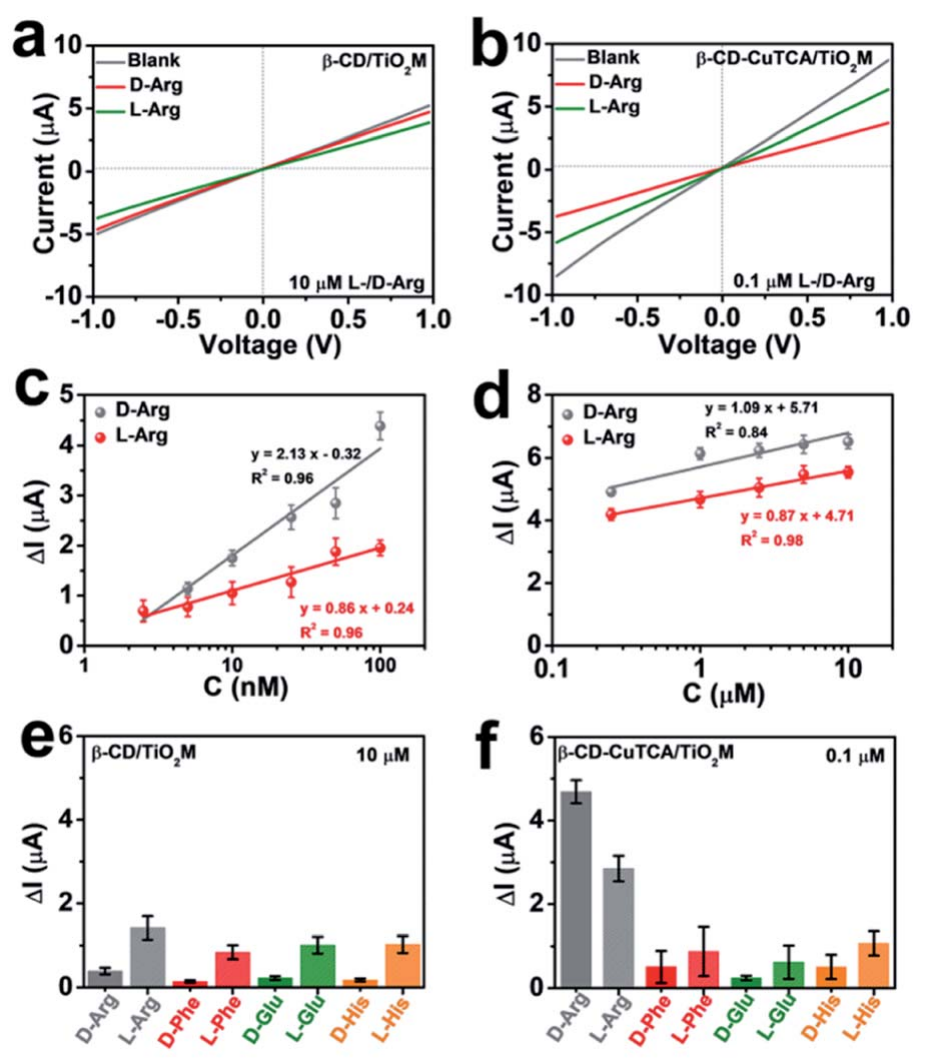
(a) *I*–*V* curves for sensing 10 μM l/d-Arg of β-CD/TiO_2_M. (b) *I*–*V* curves for sensing 0.1 μM l/d-Arg of β-CD–CuTCA/TiO_2_M. (c) The ionic current changes (Δ*I*) at +1.0 V for sensing different concentrations (2.5–100 nM) of l/d-Arg. (d) The ionic current changes (Δ*I*) at +1.0 V for sensing different concentrations (0.25–10 μM) of l/d-Arg. (e) Ionic current changes (Δ*I*) of β-CD/TiO_2_M at +1.0 V towards different chiral molecules. The concentration of each chiral molecule is 10 μM. (f) Ionic current changes (Δ*I*) of β-CD–CuTCA/TiO_2_M at +1.0 V towards different chiral molecules. The concentration of each chiral molecule is 0.1 μM. The electrolyte for electrochemical measurements contains 0.5 mM KCl and 1.0 mM H_2_O_2_ at pH 4. Error bars indicate the standard deviation of triplicate tests.

In the resulting nanochannel-based sensing device, the enantioselectivity is believed to originate from the β-CD based recognition zone. As shown in Fig. 4a, β-CD/TiO_2_M also exhibits a change in the ionic current in the presence of 10 μM l-/d-Arg with a larger current change for l-Arg. However, it is difficult to obtain a distinct response for the ionic flux when the Arg concentration is lower than this concentration (10 μM). Fig. 4b shows the ionic currents of β-CD–CuTCA/TiO_2_M for sensing 0.1 μM l/d-Arg. Obviously, CuTCA modification is helpful for amplifying the ionic current response. Compared to the ionic currents monitored on β-CD/TiO_2_M, it should be noted that β-CD–CuTCA/TiO_2_M exhibits current responses to d-Arg and l-Arg in a reverse direction mode. For example, a larger current decrease was observed for d-Arg when β-CD–CuTCA/TiO_2_M was used. In this case, this current decrease originates from the Cu(i) generation by Cu(ii) reduction. The larger affinity between l-Arg and β-CD enables more l-Arg molecules to be captured in the recognition zone than d-Arg. As a result, more d-Arg molecules arrived in the CuTCA based quantification zone and reduced Cu(ii) to Cu(i), thus leading to a more substantial decrease in the ionic current. In contrast, when more l-Arg molecules were captured in β-CD/TiO_2_M, the greater steric hindrance thus resulted in a larger decrease of ionic current. FTIR spectroscopy was employed to investigate the reaction between CuTCA and NO. The FTIR spectra display two new bands at 1683 and 1763 cm^−1^ (Fig. S17†), which can be ascribed to the anti-symmetric and symmetric N–O stretching of Cu(i)–NO adducts.^48^ These results indicate that some of the Cu(ii) centers were reduced to Cu(i) by NO.

The sensing performance of the as-proposed β-CD–CuTCA/TiO_2_M was further evaluated from the *I*–*V* curves in the presence of Arg enantiomers. Specifically, on the CuTCA side of the membrane (quantification zone), the half-cell was filled with 0.5 mM KCl solution containing 1.0 mM H_2_O_2_; on the β-CD side of the membrane (recognition zone), the half-cell was filled with 0.5 mM KCl solution containing the target enantiomers. Fig. S18a and b† show the transmembrane ionic currents of β-CD–CuTCA/TiO_2_M in the presence of different concentrations of d-Arg and l-Arg (2.5–100 nM), respectively. The ionic current changes (Δ*I*, defined as Δ*I* = *I*_0_ − *I*, where *I*_0_ and *I* are defined as the ionic current at +1.0 V, derived from the *I*–*V* curves recorded in an electrolyte containing H_2_O_2_ after 30 min of reaction) at different d/l-Arg concentrations are shown in Fig. 4c. Although the Δ*I* values increased with Arg concentration from 2.5 to 100 nM, the current changes induced by d-Arg are clearly larger than that of l-Arg at the same concentrations, suggesting that fewer l-Arg molecules reached the quantification zone. These results are consistent with the directional diffusion results (Fig. 3b), verifying that the β-CD based recognition zone has a stronger affinity with l-Arg than d-Arg. The resulting β-CD–CuTCA/TiO_2_M exhibits a good linear response to Arg sensing over the range of 2.5–100 nM (Fig. 4c). The limit of detection (LOD) was estimated to be 0.7 nM using a 3SD/*L* method (SD is the standard deviation of control, and *L* is the slope of the calibration curve). Notably, when a high concentration of target enantiomers (0.25–10 μM) was applied, the Δ*I* values of d- or l-Arg exhibit smaller differences at the same concentration (Fig. 4d and S18†), which can be attributed to a saturated adsorption state of d- and l-Arg in the recognition zone. In this case, most of the recognition sites provided by the β-CD zone were rapidly occupied. Compared with most of the recent reports on l/d-Arg recognition (Table S1†), this enantiomer sensing device showed obvious advantages, such as easy operation, low cost, and a lower LOD value.^49–52^

To demonstrate the chiral selectivity of the as-proposed asymmetric membrane for identifying Arg enantiomers, the recognition performance for other enantiomers, *i.e.*, l/d-glutamic acid (l/d-Glu), l/d-phenylalanine (l/d-Phe), and l/d-histidine (l/d-His) was evaluated. For comparison, the chiral recognition ability of β-CD/TiO_2_M was also tested (Fig. 4e, S19 and S20†). The *I*–*V* curves of β-CD/TiO_2_M showed ignorable variation when the concentration of these enantiomers was 0.1 μM (Fig. S19†), indicating poor sensitivity. When the enantiomer concentration was increased to 10 μM, β-CD/TiO_2_M samples exhibited similar recognition ability for all the enantiomer groups (Fig. S20†) − a larger ionic current variation was found for all the left chiral enantiomers (Fig. 4e). In contrast, the β-CD–CuTCA/TiO_2_M exhibited a remarkable difference response while sensing l/d-Arg (Fig. S21†). As shown in Fig. 4f, compared with l/d-Arg, the ionic currents recorded for Glu, Phe, and His enantiomers show smaller changes. Furthermore, while applying for 0.1 μM Arg enantiomer recognition, the Δ*I* value recorded on β-CD–CuTCA/TiO_2_M (Fig. 4f) is 147 times higher than that of β-CD/TiO_2_M (Fig. S19†). These results verified the excellent enantioselectivity and sensitivity of β-CD–CuTCA/TiO_2_M upon the recognition of l/d-Arg, and the remarkable sensing performance can be ascribed to the cascade of the recognition zone and quantification zone along the TiO_2_ nanochannels. Another important criterion to evaluate the enantiomer sensing platform is the stability. The tolerance of CuTCA under the experimental conditions was investigated by XRD patterns (Fig. S22†). Compared with the freshly prepared CuTCA and CuTCA/TiO_2_M, the samples show satisfactory stability after immersion in an aqueous KCl (0.5 mM, pH 4) solution for 12 h.

### Nanofluidic simulations to obtain insight into the mechanism of signal magnification

**Fig. 5:**
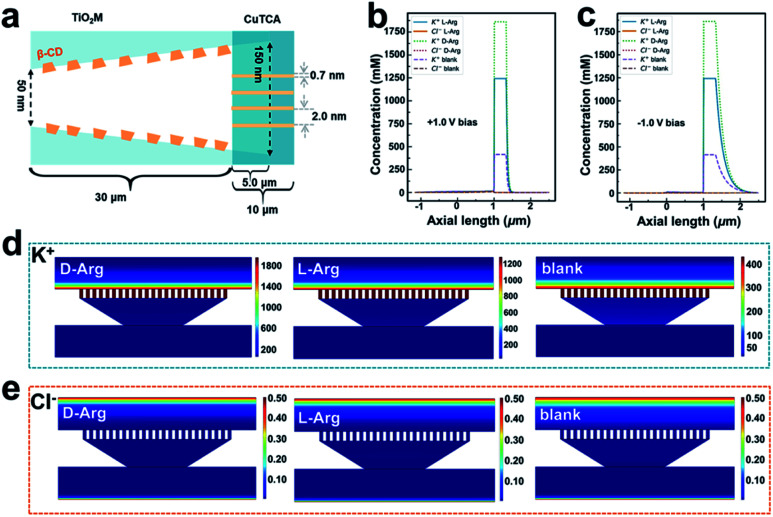
(a) Calculation model of the 2D computation domain for the heterochannels. Note that the figure is not drawn to scale. Simulated ionic concentration profiles in the β-CD–CuTCA/TiO_2_M membrane in the absence of Arg and the presence of 0.1 μM l-Arg or d-Arg when the applied voltage is +1.0 V (b) and −1.0 V (c). Simulated ionic flux images of (d) K^+^ and (e) Cl^−^ ions in the CuTCA based sensing zone for sensing 0.1 μM l-/d-Arg (the transmembrane voltage was set as −1.0 V).

It is well known that the ion transport behavior can be regulated by the surface charge of the nanochannels.^53,54^ To explore the sensing mechanism, *i*–*t* curves of β-CD–CuTCA/TiO_2_M at +1.0 V (the evolution of ionic current with reaction time, Fig. S23†) in the presence of 0.1 μM l-/d-Arg and the corresponding zeta potentials of the membranes before and after the recognition reaction (Fig. S24†) were measured. According to these data, a theoretical model (Fig. 5a, S25 and Table S2†) based on the finite element method (FEM) combined with Poisson and Nernst–Planck (PNP) equations^55,56^ (please see the Experimental section in the ESI†) was employed to simulate the Arg sensing process in β-CD–CuTCA/TiO_2_M. To calculate the ion distribution, the Comsol Multiphysics 5.5 was used with the “electrostatics (Poisson equation)” and “Nernst–Planck without electroneutrality” modules. As schematically presented in Fig. 5a, the CuTCA nanocrystal-based recognition zone is located at the base-entrance side of TiO_2_M. The pore size of CuTCA is ∼2.0 nm^37^ and the spacing between the holes of CuTCA is 0.7 nm.^57^ The thickness of the recognition zone is determined as 10 μm based on the SEM image in Fig. 1d. Fig. 5b–e show the distribution of K^+^ and Cl^−^ concentrations at the surface of the sensing zone of β-CD–CuTCA/TiO_2_M for 0.1 μM l-/d-Arg sensing. Since the CuTCA-based sensing zone carries negative surface charges, the strong electrostatic interactions will attract more K^+^ ions into the CuTCA/TiO_2_M nanochannel, resulting in the accumulation of K^+^ ions in the CuTCA/TiO_2_M nanochannel at a bias of +1.0 V (Fig. 5b) and −1.0 V (Fig. 5c). The accumulated K^+^ ions in the nanochannels of CuTCA/TiO_2_M result in an increased ion conductance of CuTCA/TiO_2_M. Fig. 5d shows the concentration profiles of K^+^ ions at −1.0 V in the CuTCA/TiO_2_M nanochannel for l-/d-Arg sensing and the blank solution. Compared to the blank solution and l-Arg, the K^+^ flux in the sensing zone shows an obviously high intensity for d-Arg sensing, which can be attributed to the lower affinity between the β-CD based recognition zone and d-Arg. These results are consistent with the experimental ionic current changes in Fig. 4 (the Δ*I* values induced by d-Arg are larger than those of l-Arg at the same concentrations). Owing to the strong electrostatic repulsion, it is difficult for Cl^−^ ions to enter the negatively charged CuTCA/TiO_2_M. As a result, the Cl^−^ concentration in the sensing zone is very low for l-Arg and d-Arg sensing, and the ionic flux intensity is similar to that for the blank solution (Fig. 5e).

### Fluorescence sensing ability for l/d-Arg discrimination

Besides the electrochemical signal from the *I*–*V* curves of nanochannels, the appearance of a solid-state fluorescence emission in Arg sensing provides a visual method for quantification. Owing to the π–π* transition of the triphenylamine group,^37^ the TCA ligand has a strong photoluminescence at ∼430 nm when excited at 350 nm. Attributed to the quenching effect of the paramagnetic center, the fluorescence emission of TCA disappeared by the coordinating reaction with Cu(ii) to form CuTCA. It has been discovered that the diamagnetic species Cu(i) can alleviate fluorescence quenching caused by paramagnetic Cu(ii) ions.^37^ Therefore, a recovery of fluorescence is expected to be observed on the as-prepared asymmetric membrane after the Cu(ii) in CuTCA was reduced to Cu(i) using NO. To estimate the visual sensing possibility, the solid-state fluorescence spectra of β-CD–CuTCA/TiO_2_M in the presence of different concentrations of d-Arg and l-Arg were recorded. As shown in Fig. S26a and b,† the fluorescence emission appeared in the presence of d/l-Arg enantiomers, and the intensity increased with increasing Arg concentration. Fig. S26c† shows the relationship between l/d-Arg concentrations and fluorescence intensities. The membranes show distinct differences in fluorescence intensities when sensing Arg enantiomers from 0.1 μM to 10 μM. Compared to l-Arg, larger fluorescence emissions were observed for the same concentration of d-Arg, which can be explained by the high affinity between β-CD and l-Arg inducing less l-Arg in the quantification zone. To study the possibility of application as a visual screening platform, the fluorescence images of the membrane for sensing a series of concentrations of Arg enantiomers were recorded (Fig. S26d†). Apparently, the fluorescence became more obvious with increasing Arg concentration. Notably, the lowest d-Arg concentration for a visual fluorescence is only 0.1 μM, whereas a visual fluorescence requires 2.5 μM l-Arg. The solid-state fluorescence recovery of the as-proposed β-CD–CuTCA/TiO_2_M can also serve as a visual platform for the qualitative discrimination of Arg enantiomers.

## Experimental

### Preparation of TiO_2_M

TiO_2_ nanochannel membranes were grown from Ti foils (15 mm × 15 mm × 0.1 mm) by electrochemical anodization. For this purpose, the Ti foils were first sequentially rinsed with acetone, ethanol, and deionized water and then dried in air. Anodization was carried out in a mixture of ethylene glycol/lactic acid/water electrolyte containing 0.1 M NH_4_F at 120 V and 150 V for 20 min and 2 min, respectively. To obtain an open-ended TiO_2_ nanochannel membrane, the obtained samples were dipped in H_2_O_2_ (30%) until the titanium substrates were separated from the membrane. The prepared samples were annealed at 450 °C for 2 h in air with a heating rate of 3 °C min^−1^.

### Preparation of CuNPs/TiO_2_M

CuNPs/TiO_2_M was assembled in a home-made H-type cell. The membrane was placed between the two cells of the homemade electrolyte cell, and a quartz window was set on one side of the cell (base side of TiO_2_M) for allowing UV light to pass through and reach the TiO_2_M surface. One half of the cell (tip side of TiO_2_M) was filled with 5.0 mM Cu(CH_3_COO)_2_·H_2_O. Another half of the cell (base side of TiO_2_M) was filled with pure water. When driven under +1.0 V for 120 min, the CuNPs migrated and then deposited on the base side of the nanochannel exposed to UV light (3 W LED, 365 nm).

### Preparation of CuTCA/TiO_2_M

The Cu-MOF-modified TiO_2_M was prepared using H_3_TCA as the organic ligand and the anchored CuNPs as the precursor of Cu^2+^.^37,58^ Briefly, 10 mg of H_3_TCA was dissolved in 10 mL of a mixture of DMF and CH_3_OH (*V*_DMF_ : *V*_CH_3_OH_ = 1 : 1). The solution was sonicated for 30 min and then transferred to a 50 mL Teflon-lined autoclave. The as-formed CuNPs/TiO_2_M (diameter 10 mm) was added to the aforementioned solution. The sealed vessel was then held at 120 °C for 48 h to grow CuTCA in TiO_2_M. The resulting CuTCA/TiO_2_M sample was carefully washed with DMF and CH_3_OH to remove the unreacted ligands in the nanochannels and then dried in an 80 °C vacuum oven to remove the remaining solvent.

### Preparation of bulk CuTCA

A mixture of H_3_TCA (94 mg, 0.25 mmol) and Cu(NO_3_)_2_ 6H_2_O (242 mg, 1 mmol) was dissolved in 15 mL of a mixture of DMF and CH_3_OH (*V*_DMF_ : *V*_CH3OH_ = 1 : 1). The solution was sonicated for 30 min and then transferred to a 50 mL Teflon-lined autoclave. The sealed vessel was kept in an oven at 120 °C for 48 h. The resulting green crystals were carefully washed with DMF and CH_3_OH to remove the unreacted ligands and then dried in an 80 °C vacuum oven to remove the remaining solvent.

### Preparation of β-CD–CuTCA/TiO_2_M

The diffusion method was used for β-CD modification in CuTCA/TiO_2_M in a home-made H-type cell. One half of the cell (tip side of CuTCA/TiO_2_M) was filled with 1.0 mM PP. Another half of the cell (base side of TiO_2_M) was filled with pure water to diffuse for 24 h. Then, 1.0 mM β-CD solution was used for self-assembly with PP.

### Detection of chiral arginine by electrochemical measurements

Chiral arginine was detected in a home-made H-type cell. A pair of homemade Ag/AgCl electrodes was used to measure the resulting ionic current. The membrane was mounted between two halves of the conductance cell. One half of the cell was filled with 0.5 mM KCl and chiral arginine solutions with different concentrations. Another half of the cell was filled with 0.5 mM KCl and 1.0 mM H_2_O_2_. The effective membrane area for *I*–*V* property measurements is 3.14 mm^2^. Linear sweep voltammetry (LSV) was carried out from −1.0 V to 1.0 V at a scan rate of 50 mV s^−1^.

## Conclusions

In summary, an enantioselective sensing platform for Arg enantiomers with high sensitivity was constructed based on asymmetric TiO_2_M. Benefiting from the *I*–*V* properties of the nanochannel structure and the cascade recognition–quantification zone design along the TiO_2_ nanochannels, the resulting β-CD–CuTCA/TiO_2_M exhibited a sensitive and selective performance for the discrimination of Arg enantiomers. Besides providing the *I*–*V* signal, the as-proposed membrane can also act as a visual fluorescence platform for a convenient and rapid discrimination of Arg enantiomers. This work not only paves a new way to design asymmetric nanochannels, but also verifies that the cascade of recognition–quantification zones is an effective strategy to achieve selective and sensitive recognition of enantiomers.

## Data availability

The data supporting the findings of this study are available within the article and in the ESI.†

## Author contributions

Y.-Y. Song conceived the concept and directed the project. J. L. Guo, X. J. Xu, and J. J. Zhao performed the experiments. Z.-Q. Wu carried out the theoretical study. Z. D. Gao collected and analyzed the data. J. L. Guo prepared the first draft of this manuscript, and all the authors modified the manuscript.

## Conflicts of interest

There are no conflicts to declare.

## Supplementary Material

SC-013-D2SC03198A-s001
