# Supplementary material for: Locally superengineered cascade recognition–quantification zones in nanochannels for sensitive enantiomer identification
Source: Chem Sci. 2022 Aug 8;13(34):9993–10002. doi: 10.1039/d2sc03198a (PMC9430310; doi:10.1039/d2sc03198a)
Supplement: SC-013-D2SC03198A-s001 [file SC-013-D2SC03198A-s001.pdf]

Electronic Supplementary Material (ESI) for Chemical Science.
This journal is © The Royal Society of Chemistry 2022

Supplementary Information

Locally superengineered cascade recognition-quantification zones in nanochannels for sensitive enantioselective identification

Junli Guo,[a] Huijie Xu,[a] Junjian Zhao,[a] Zhida Gao,[a] Zeng-Qiang Wu,*[b] and Yan-Yan Song*[a]

[a]College of Sciences, Northeastern University, Shenyang 110819, China

[b]School of Public Health, Nantong University, Nantong, 226019, China

Corresponding Authors

E-mail: yysong@mail.neu.edu.cn

E-mail: zqwu@ntu.edu.cn

EXPERIMENTAL SECTION

Chemicals and reagents. Ti sheets (0.1 mm thickness, 99.6% purity) were purchased from Baosheng Hardware (Bao ji). 4,4',4''-Tricarboxytriphenylamine (H_3TCA), riboflavin sodium phosphate (RFMP), Rhodamine B (RhB), Cupric acetate monohydrate ($Cu(CH_3COO)_2 \cdot H_2O$), β-Cyclodextrin (β-CD), Phenyl phosphate disodium salt ($C_6H_5Na_2O_4P$), L-arginine (L-Arg) and D-arginine (D-Arg) were purchased from Aladdin. Ammonium fluoride (NH_4F), ethylene glycol, N,N-dimethylformamide (DMF, 99.8%), methanol (CH_3OH), and other chemicals were purchased from Sinopharm Chemical Reagent and used as received without further purification. All aqueous solutions were prepared using Millipore Milli-Q water with a resistivity of 18.0 MΩ·cm.

Apparatus. Morphology was characterized using a field-emission scanning electron microscope (Hitachi SU8000, Japan). A JEOL 2000FX high-resolution transmission electron microscope (HRTEM) (Japan) was used to record TEM and HRTEM images. Crystal structures were identified by XRD acquired using an X’Pert XRD spectrometer (Philips, USA) using a CuKα X-ray source. Zeta potential was measured using a Zetasizer Nano ZS90 analyzer (Malvern, USA). X-ray photoelectron spectra (XPS) were recorded using a Perkin-Elmer Physical Electronics 5600 spectrometer using Al Kα radiation at 13 kV as the excitation source. The takeoff angle of emitted photoelectrons was 45°, and the binding energy of target elements was determined at a pass energy of 23.5 eV with a resolution of 0.1 eV. The binding energy of Ti 2p signal (458.0 eV) was used as the reference. The content of copper element was determined by inductively coupled plasma-optical emission spectroscopy (ICP-OES) using a PerkinElmer spectrometer (Avio 500, USA). UV-vis absorption spectra were recorded using a Perkin–Elmer spectrometer (Lambda 750S, USA). Fluorescence spectra were recorded using an F-7000 fluorescence spectrophotometer (Hitachi, Japan) with a scan speed of 2400 nm min^{-1}. FTIR spectroscopy was performed using a Nicolet 6700 instrument (Thermo Fisher, USA). Fluorescence imaging was performed using an upright fluorescence microscope (BX53M). A CHI660D electrochemical workstation (CH Instrument, USA) was used for all electrochemical tests equipped with two Ag/AgCl electrodes as the anode and cathode. Photocatalytic reactions were performed under the irradiation of an ultraviolet LED (3 W, central wavelength at ~365 nm).

Numerical simulations for ionic transport in β-CD-CuTCA/TiO_2M nanochannel array. The nanofluidic sensor mechanism for Arg was analyzed by a theoretical model and was performed on the finite element method (FEM) combining with Poisson and Nernst-Planck (PNP) equations. The equations are shown below:[S1,S2]

$$\nabla^2\varphi = -\frac{F}{\varepsilon}\sum z_i c_i \tag{S1}$$

$$j_i = D_i(\nabla c_i + \frac{z_i F c_i}{RT}\nabla\varphi) \tag{S2}$$

$$\nabla \cdot j_i = 0 \tag{S3}$$

where j_i, D_i, C_i, φ, R, F, T, and ε are the ionic flux, diffusion coefficient, ion concentration, electrical potential, the universal gas constant, Faraday constant, absolute temperature, and dielectric constant of the electrolyte solution, respectively. The electric potential and ionic concentration can be characterized as described by Equation S1, which is called the Poisson equation. Equation S2 is the Nernst-Planck equation which characterizes the transport behavior in the charged nanochannel. Besides, when the system reaches a steady state, the flux should satisfy Equation S3. The coupled Equation S1-S3 can be used to calculate the ion concentration distribution and can be solved by assuming appropriate boundary conditions.

$$\vec{n} \cdot \nabla\varphi = -\frac{\sigma}{\varepsilon} \tag{S4}$$

$$\vec{n} \cdot j = 0 \tag{S5}$$

The boundary condition for potential φ on the channel wall is Equation S4, where the ionic flux has zero normal components at boundaries Equation S5.

Figures

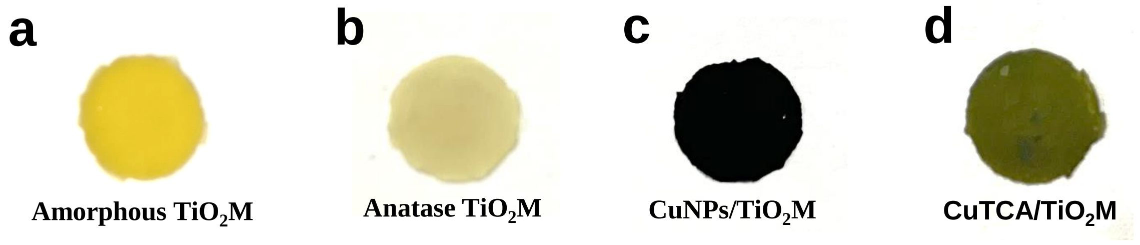

Fig. S1 Digital photos of (a) amorphous TiO_2M, (b) anatase TiO_2M, (c) CuNPs/TiO_2M and (d) CuTCA/TiO_2M.

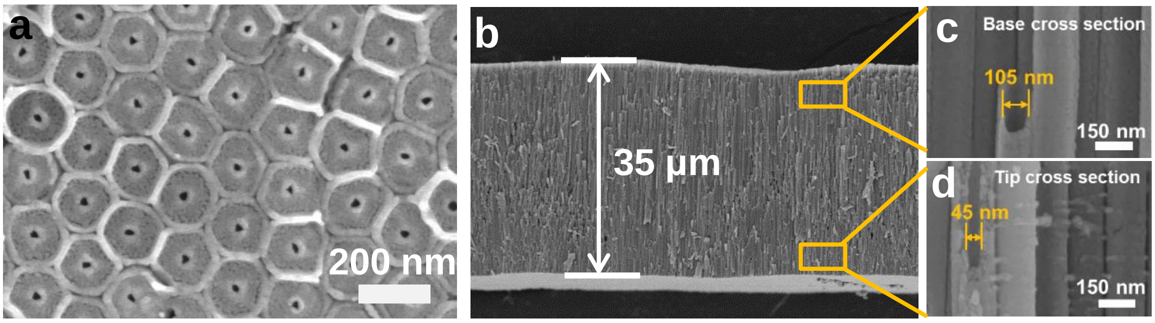

Fig. S2 SEM images of TiO_2M (a) tip entrance side view, (b) cross-sectional view, (c) base cross section view, and (d) tip cross section view.

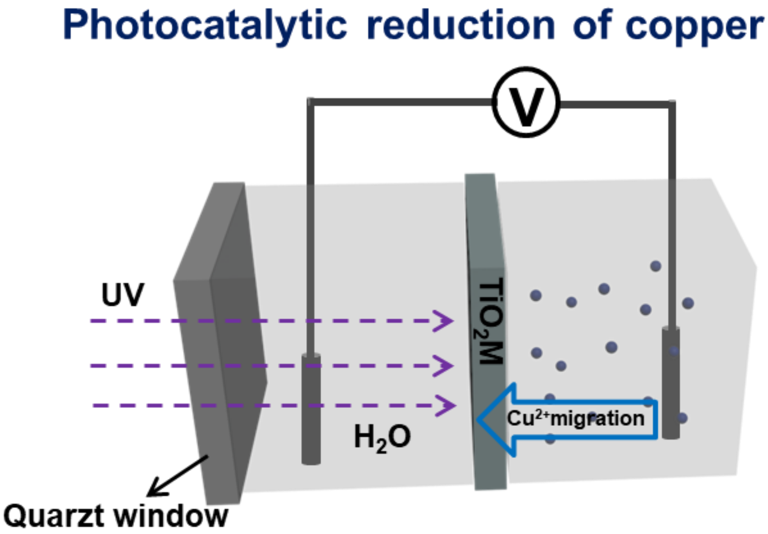

Fig. S3 Schematic diagram of the setup for the modification of CuNPs in the base side of TiO_2M (the left cell is filled with pure water, and the right cell is filled with 5.0 mM $Cu(CH_3COO)_2 \cdot H_2O$). When driven under +1.0 V for 120 min, the CuNPs migrated and then deposited into the base side of nanochannel exposed to UV light (3 W LED, 365 nm).

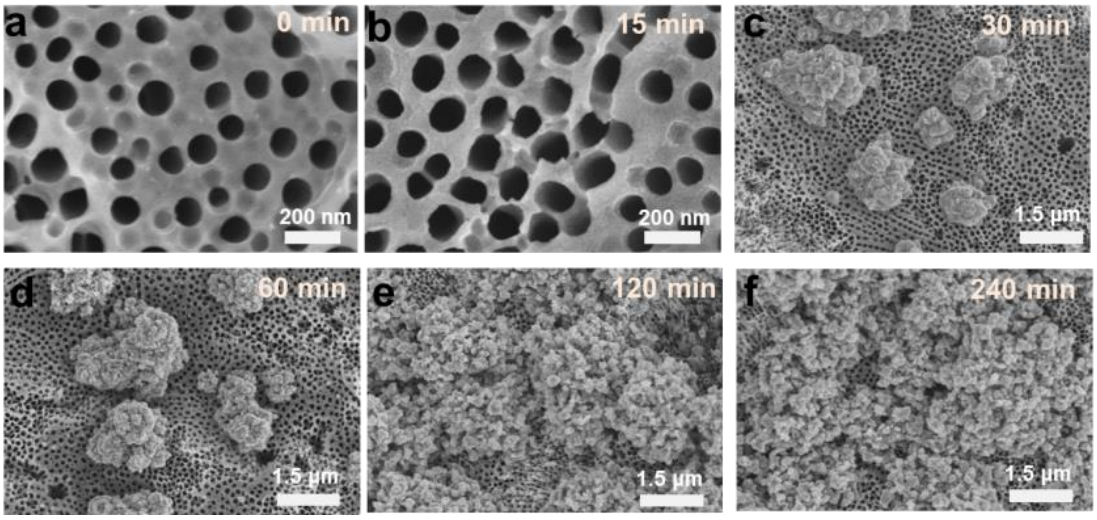

Fig. S4 Base entrance side SEM images of TiO_2M with CuNPs modified with different irradiation times: (a) 0 min, (b) 15 min, (c) 30 min, (d) 60 min, (e) 120 min, (f) 240 min (LED: 3W, 365 nm).

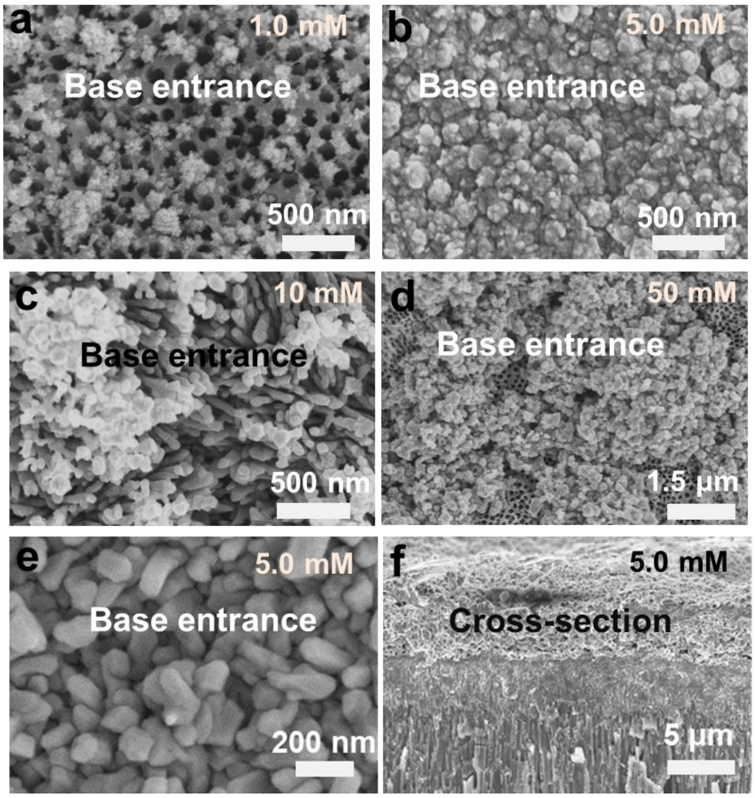

Fig. S5 Base entrance side SEM images of TiO_2M with CuNPs modified with different concentrations of copper acetate: (a) 1.0 mM, (b) 5.0 mM, (c) 10 mM, (d) 50 mM. SEM images of TiO_2M with CuNPs modified with 5.0 mM copper acetate, (e) base entrance side view, and (f) cross-section view (LED: 3 W, 365 nm; irradiation time: 120 min) .

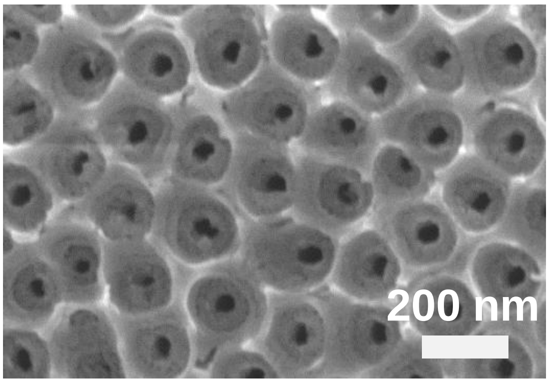

Fig. S6 Tip entrance side SEM images of CuTCA/TiO_2M.

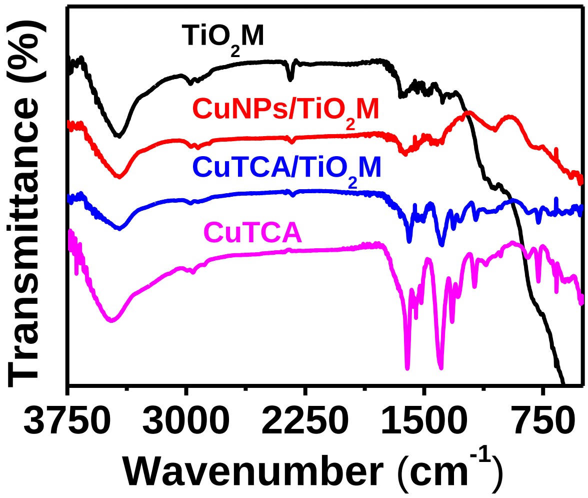

Fig. S7 FTIR spectra of TiO_2M, CuNPs/TiO_2M, CuTCA/TiO_2M, and CuTCA.

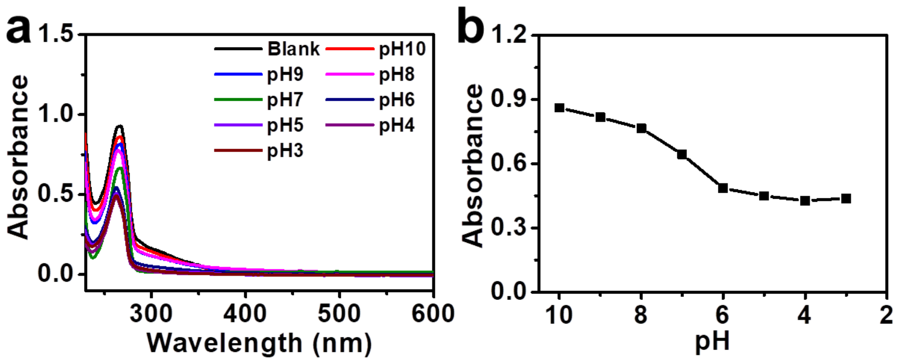

Fig. S8 Effect of solution pH on PP adsorption. (a) UV-Vis absorption spectra and (b) the absorbance intensity at 267 nm of PP in the solution after 3.0 mg TiO_2M incubation for 24 h.

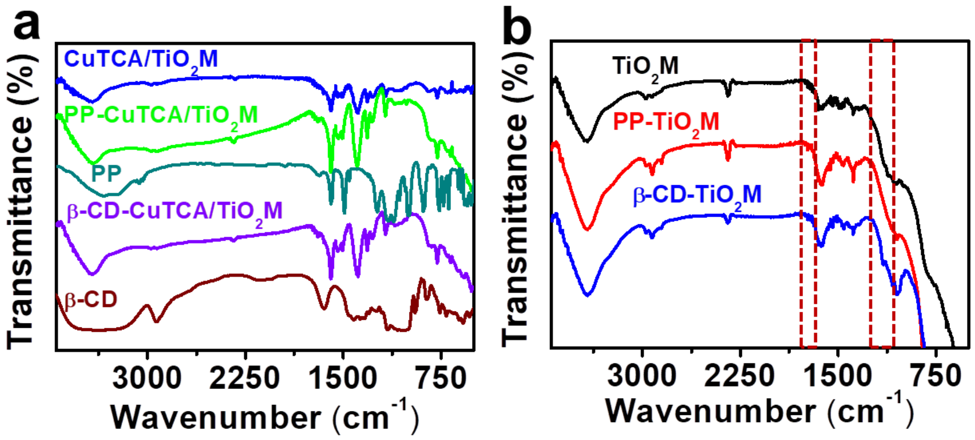

Fig. S9 FTIR spectra of (a) PP, β-CD, CuTCA/TiO_2M, and PP-CuTCA/TiO_2M with and without β-CD modification. (b) TiO_2M, PP-TiO_2M with and without β-CD modification.

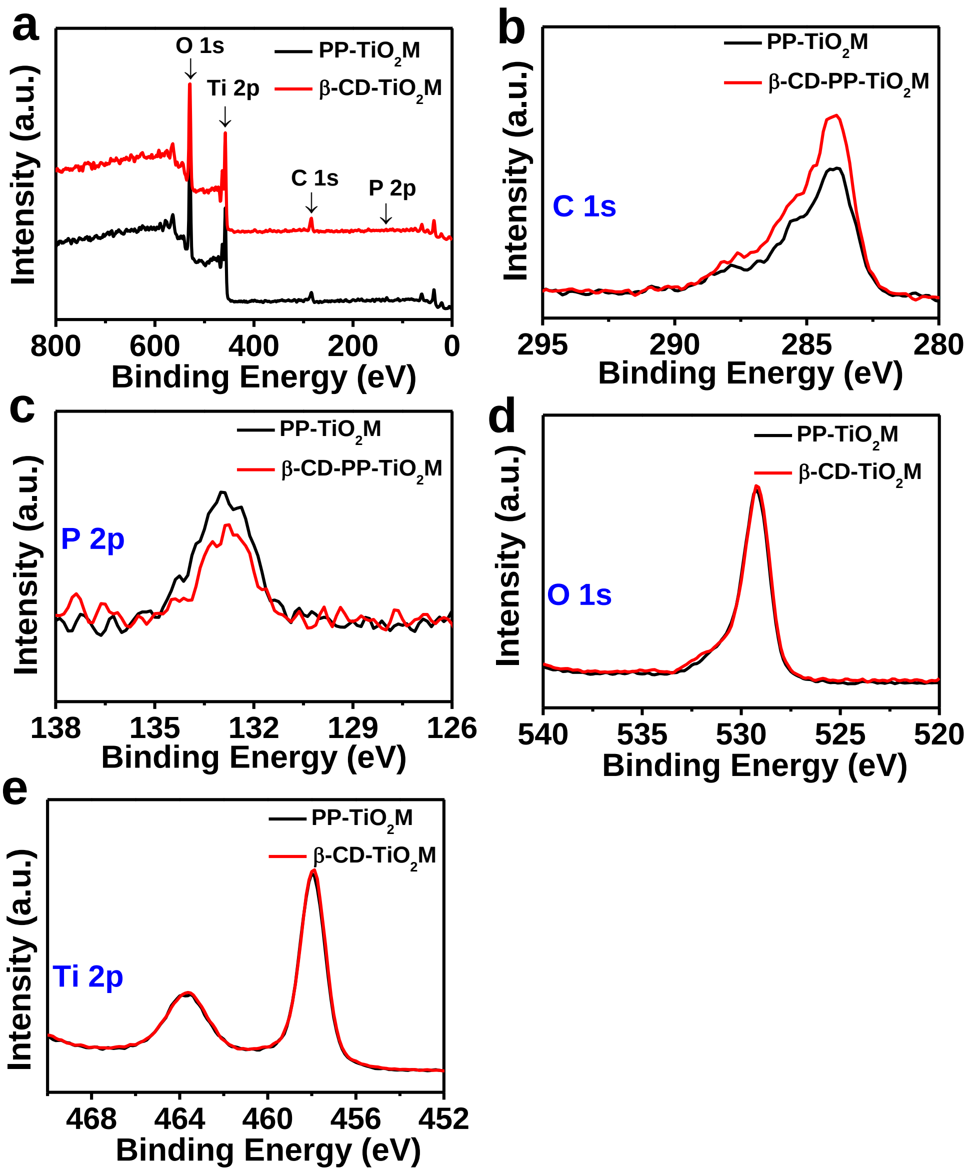

Fig. S10 XPS spectra of TiO_2M before and after β-CD modification. (a) Survey spectra, and high-resolution peaks of (b) C 1s, (c) P 2p, (d) O 1s, (e) Ti 2p signals.

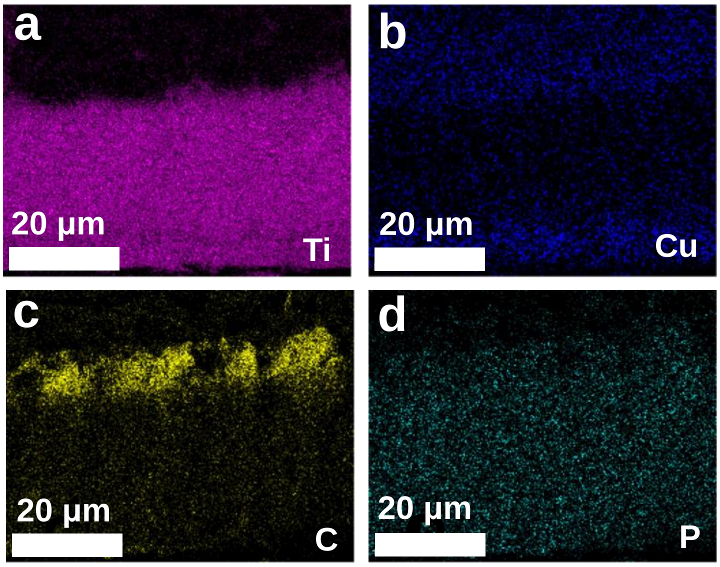

Fig. S11 Elemental mapping of β-CD-CuTCA/TiO_2M (a) Ti, (b) Cu, (c) C, and (d) P elements.

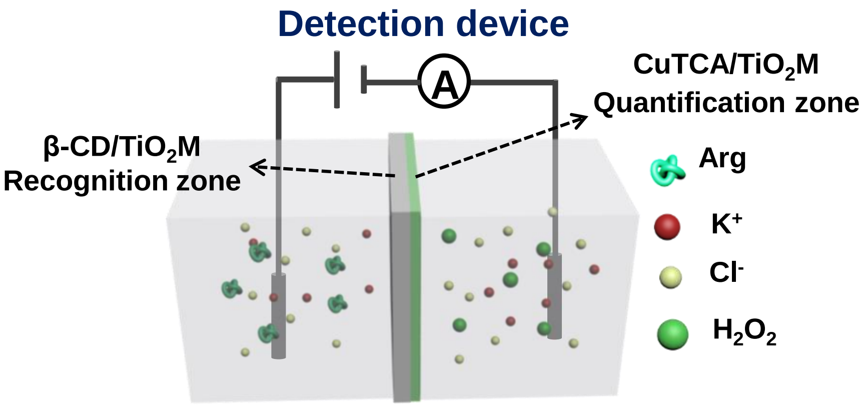

Fig. S12 Schematic diagram of setup for chiral Arg detection (the left cell is filled with KCl solution containing L-Arg or D-Arg, and the right cell is filled with KCl solution containing 1.0 mM H_2O_2).

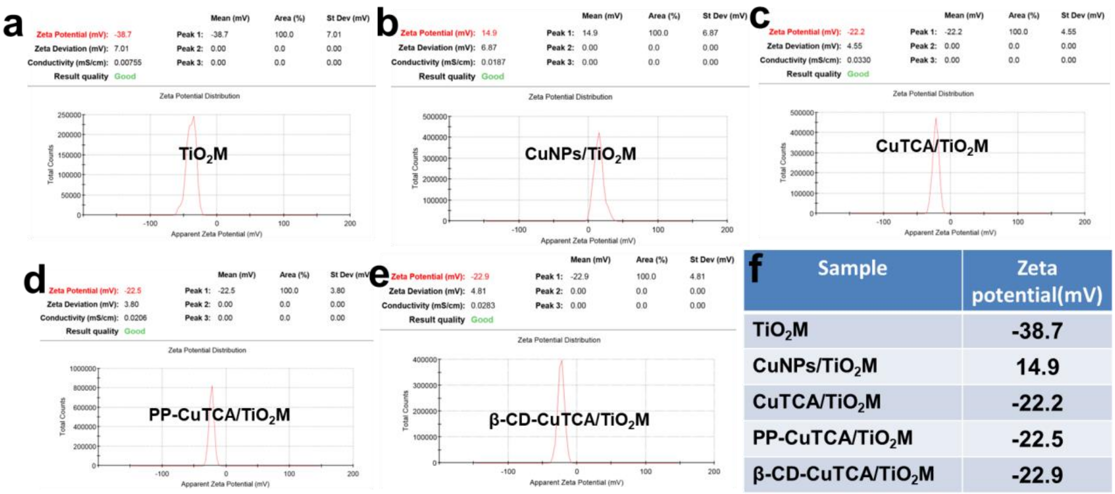

Sample	Zeta potential(mV)
TiO_2M	-38.7
CuNPs/TiO_2M	14.9
CuTCA/TiO_2M	-22.2
PP-CuTCA/TiO_2M	-22.5
β-CD-CuTCA/TiO_2M	-22.9

Fig. S13 Zeta potentials of (a) TiO_2M, (b) CuNPs/TiO_2M, (c) CuTCA/TiO_2M, (d) PP-CuTCA/TiO_2M, and (e) β-CD-CuTCA/TiO_2M. (f) Table for the Zeta potential values of the above samples.

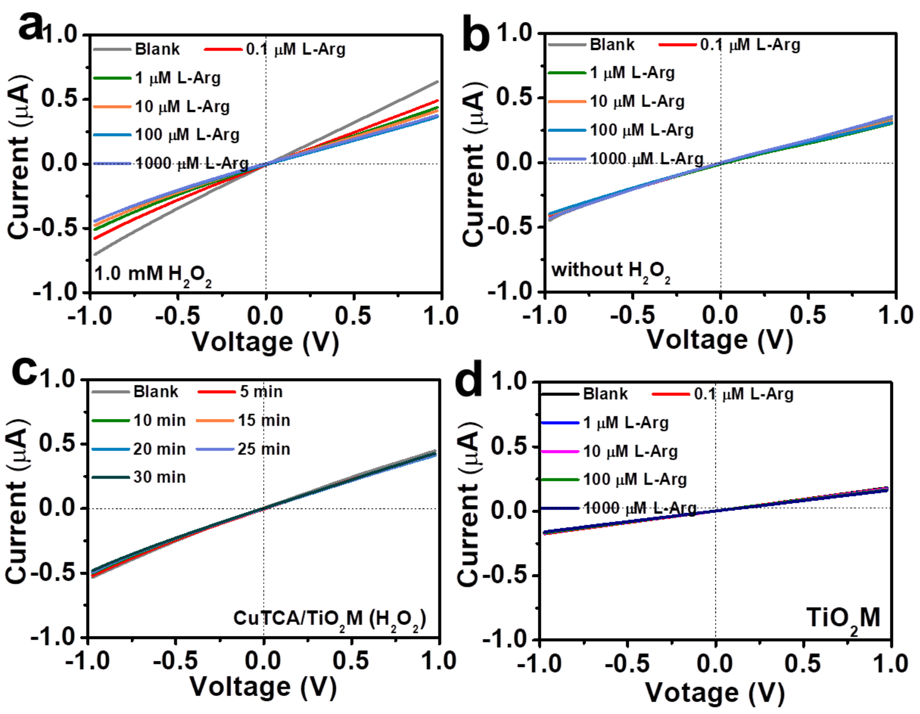

Fig. S14 (a) *I*–*V* properties of CuTCA/TiO_2M in different concentrations of L-Arg with 1.0 mM H_2O_2. (b) *I*–*V* properties of CuTCA/TiO_2M in different concentrations of L-Arg without H_2O_2. (c) *I*–*V* properties of CuTCA/TiO_2M without L-Arg in 1.0 mM H_2O_2. (d) *I*–*V* curves of TiO_2M in the presence of different concentrations of L-Arg with 1.0 mM H_2O_2.

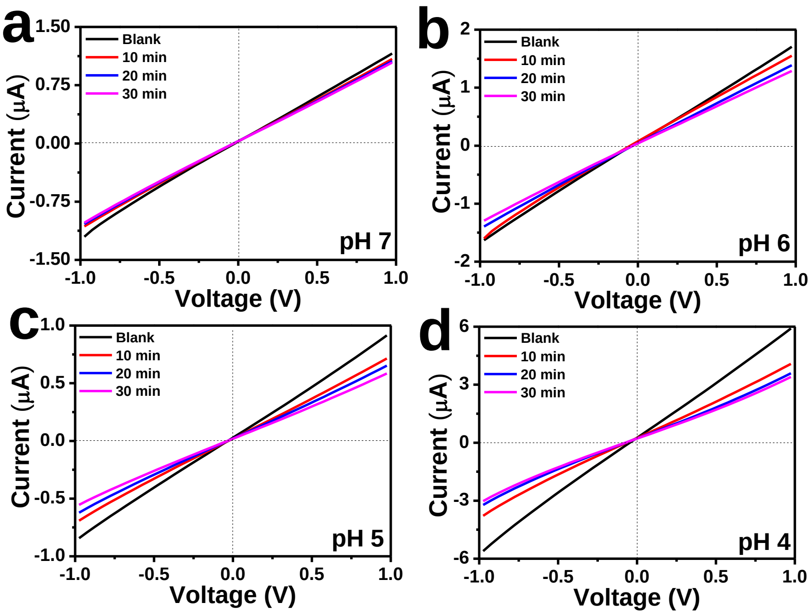

Fig. S15 *I–V* curves of CuTCA/TiO_2M for the detection of Arg at different pH values: (a) pH 7, (b) pH 6, (c) pH 5, and (d) pH 4. The electrolyte for electrochemical measurement contains 0.5 mM KCl and 0.1 μM L-Arg or 1.0 mM H_2O_2.

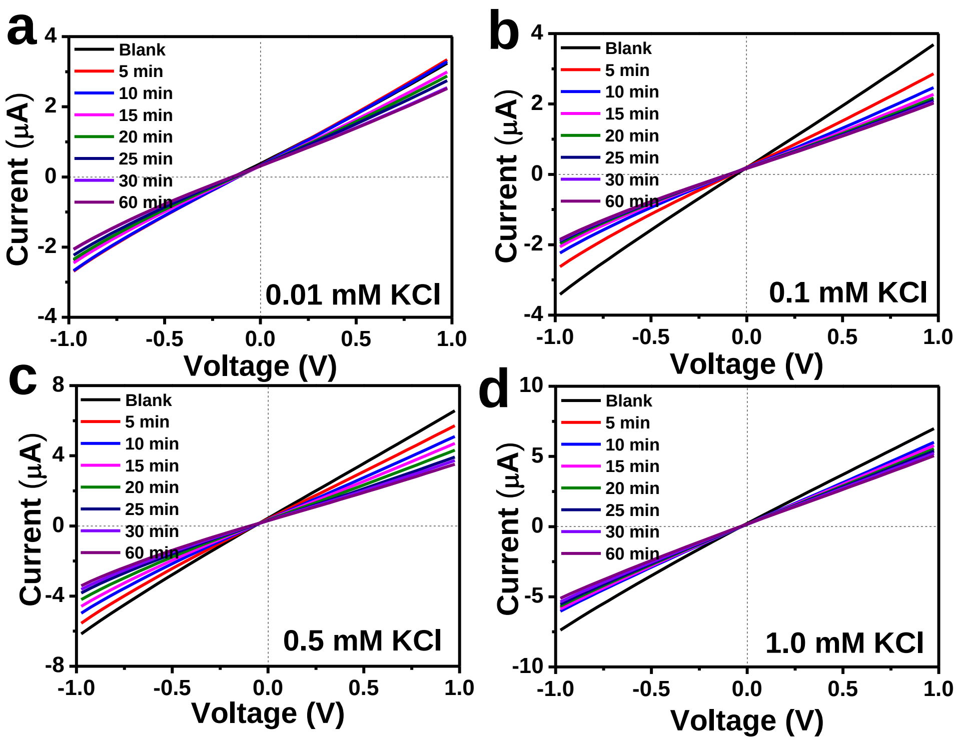

Fig. S16 *I–V* curves CuTCA/TiO_2M for the detection of Arg in pH 4 electrolyte containing different concentrations of KCl: (a) 0.01 mM (b) 0.1 mM (c) 0.5 mM, (d) 1.0 mM KCl. The electrolyte contains 0.1 μM L-Arg and 1.0 mM H_2O_2.

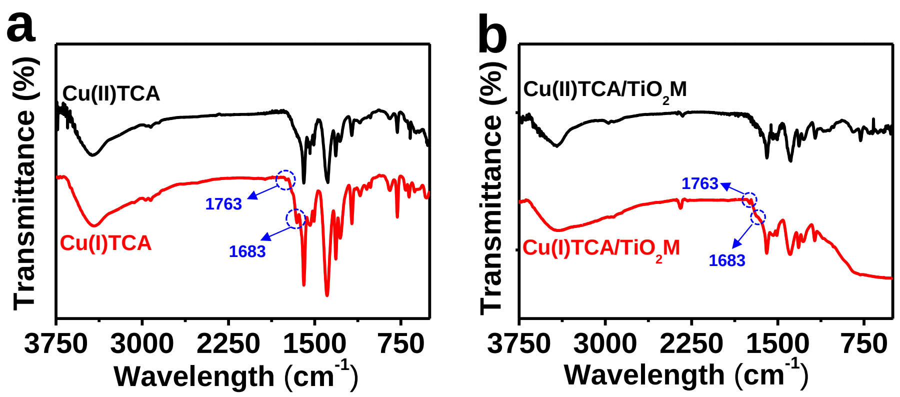

Fig. 17 FTIR spectra of (a) CuTCA powder and (b) CuTCA/TiO_2M before and after reaction with NO (produced by Arg reaction with H_2O_2).

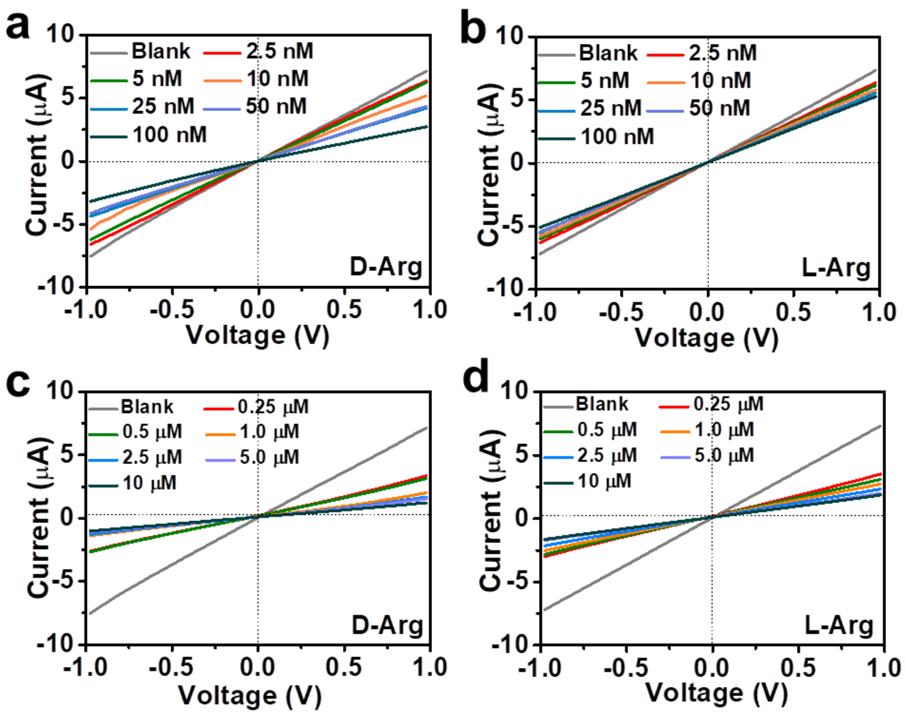

Fig. S18 Enantioselective sensing performance. *I–V* curves for sensing different concentrations (2.5-100 nM) of (a) D-Arg, (b) L-Arg. *I–V* curves for sensing different concentrations (0.25 μM-10 μM) of (c) D-Arg, (d) L-Arg. The electrolyte for electrochemical measurement contains 0.5 mM KCl and 1.0 mM H_2O_2 at pH 4.

Table S1 Comparison of various methods for L/D-Arg detection.

Method	Mechanisms	Sensitivity data	Reference
PET nanochannel modified with bovine serum albumin (BSA) based electrochemical method.	Arg contains the functional guanidine group which can interact with BSA via ionized carboxyl groups (–COO^-), forming ion pairs through cooperative interactions.	0.05 mM	S3
Tb^{3+}-functionalized MOF-based fluorescence method.	The interaction between arginine and Tb^{3+} caused intense luminescence quenching by weakening the energy transfer process between Tb^{3+} and the bridging ligands of the frameworks.	1.0 μM	S4
Carbon dots derived from reduced L-glutathione-based fluorescence method.	Electrostatic interaction and electron transfer.	28.5 nM	S5
A chiral calix[4]arene receptor (5) having (*R*)-2-phenylglycinol moiety-based quartz crystal microbalance method.	It was considered that the size-fit concept, three-dimensional structures of molecules, steric effects and interactions between moieties of the sensible film layer and analyte molecules such as hydrogen bonding interactions may play a major role in this difference.	0.38 μM	S6
A smart identification-detection cascade device based on asymmetric MOFs in nanochannel membrane based electrochemical method.	The passing D-Arg enantiomer reacts with H_2O_2 to produce NO, which can reduce Cu^{2+} to Cu^+ to change the transmembrane ionic current in nanochannels.	0.7 nM	This work

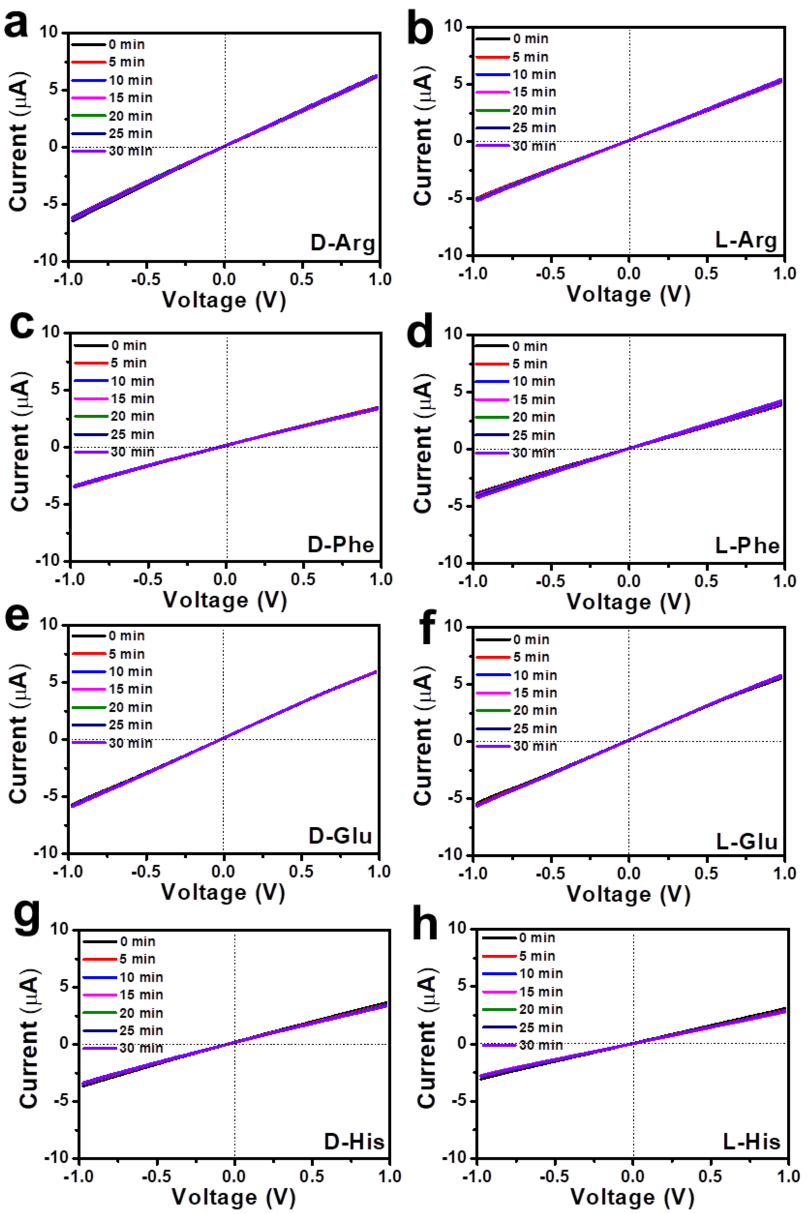

Fig. S19 *I*−*V* curves of β-CD/TiO_2M for sensing (a) D-Arg, (b) L-Arg, (c) D-Phe, (d) L-Phe, (e) D-Glu, (f) L-Glu, (g) D-His, and (h) L-His. The concentrations of all enantiomers were 0.1 μM.

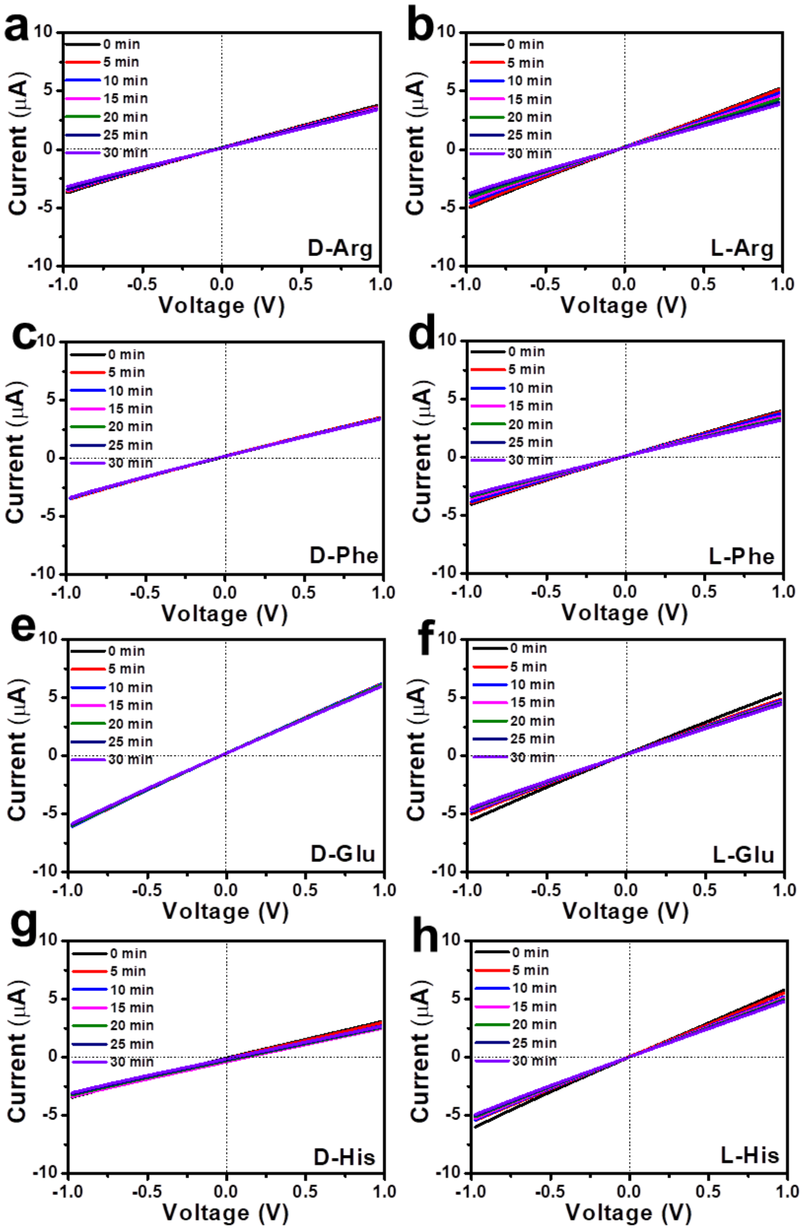

Fig. S20 *I*−*V* curves of β-CD/TiO_2M for sensing (a) D-Arg, (b) L-Arg, (c) D-Phe, (d) L-Phe, (e) D-Glu, (f) L-Glu, (g) D-His, and (h) L-His. The concentrations of all enantiomers were 10 μM.

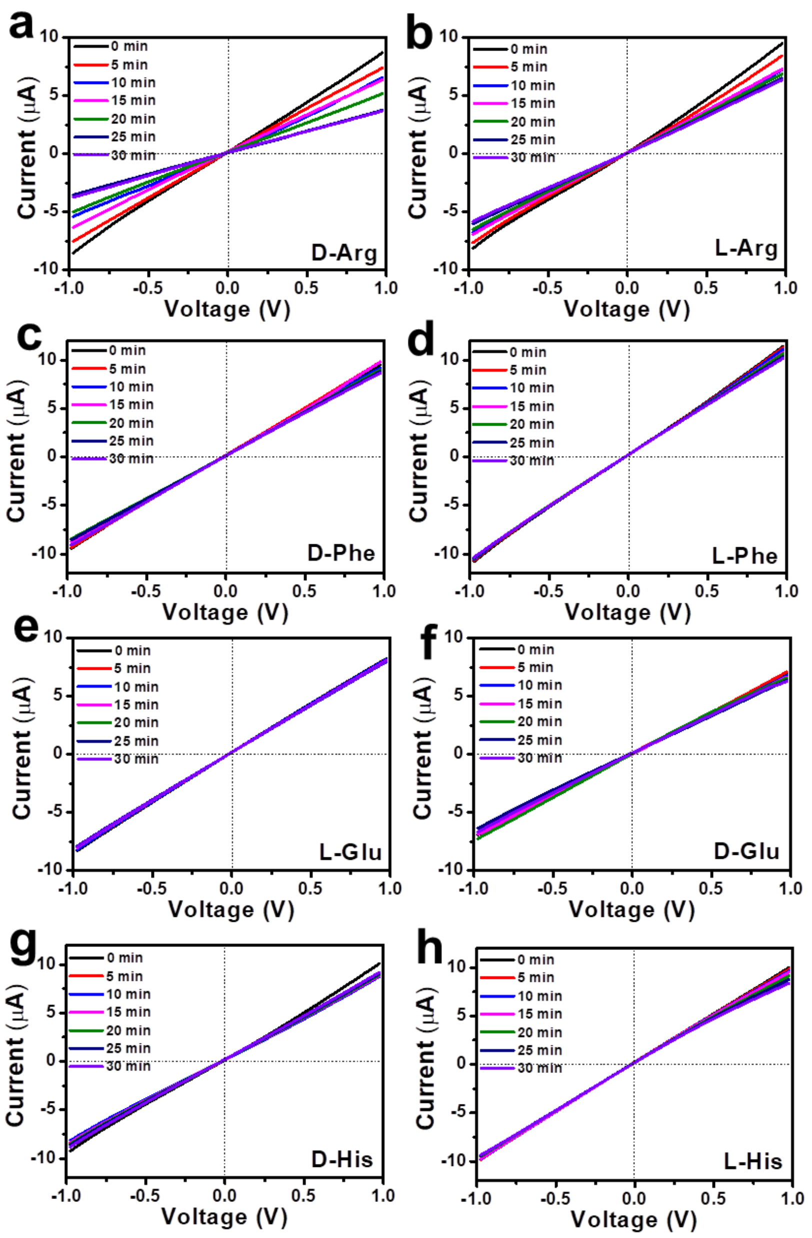

Fig. S21 I–V curves of β-CD-CuTCA/TiO_2M for sensing (a) D-Arg, (b) L-Arg, (c) D-Phe, (d) L-Phe, (e) D-Glu, (f) L-Glu, (g) D-His, (h) L-His. The concentrations of all enantiomers were 0.1 μM.

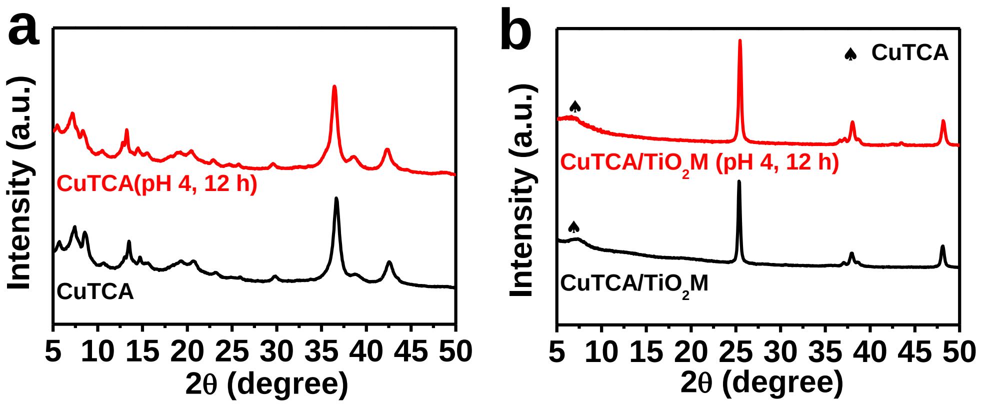

Fig. S22 XRD pattern of (a) CuTCA and (b) CuTCA/ TiO_2M before and after immersing in 0.5 mM KCl (pH 4) solution for 12 h.

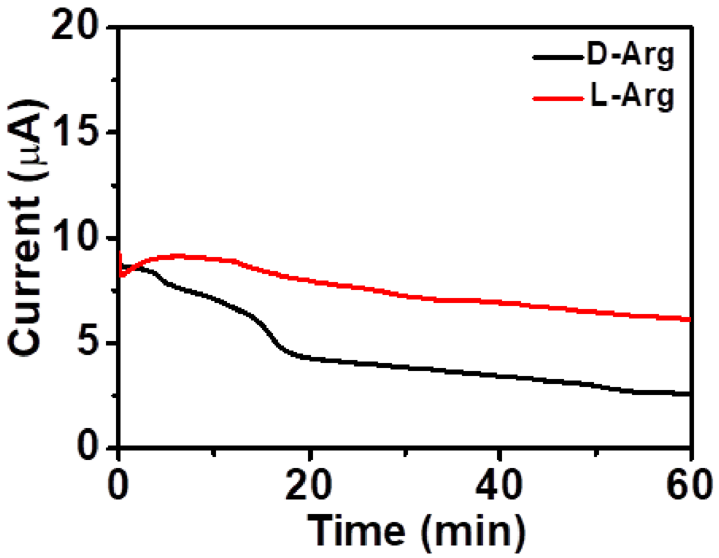

Fig. S23 Current–time response of β-CD-CuTCA/TiO_2M in 0.5 mM KCl (pH 4) electrolyte containing 0.1 μM L/D-Arg at +1.0 V.

To gain more clear insight on the selectivity of the as-proposed hybrid channels for Arg enantiomer sensing, ionic transmembrane currents of β-CD-CuTCA/TiO_2M at +1.0 V with reaction time was measured in the presence of D-Arg or L-Arg and shown in Figure S17. Compared with L-Arg, the ionic currents show a significantly decrease at ~17 min for D-Arg sensing. As the current decrease is originated from the continuously generated Cu(I)TCA in channels, these results suggest more D-Arg molecules reach the sensing zone. Notably, the ionic current reaches a plateau at ~30 min, indicating that the mass generation and diffusion reached a balanced state, consistent with the *I*–*V* curves recorded at 30 min.

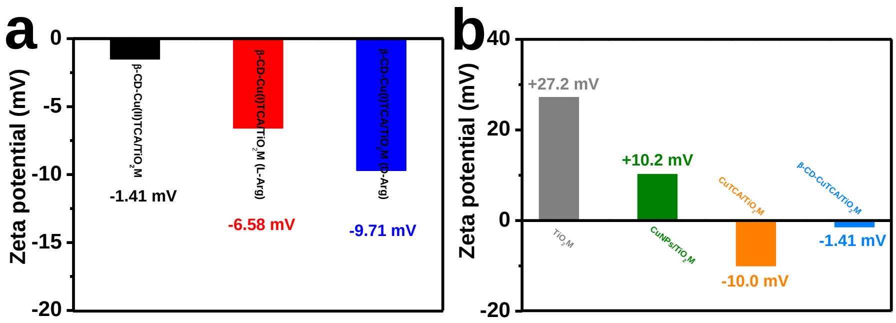

Fig. S24 (a) Zeta potential of β-CD-CuTCA/TiO_2M before and after sensing 0.1 μM D-Arg or L-Arg. (b) Zeta potential of TiO_2M, CuNPs/TiO_2M, CuTCA/TiO_2M and β-CD-CuTCA/TiO_2M. The solvent is 0.5 mM KCl (pH 4).

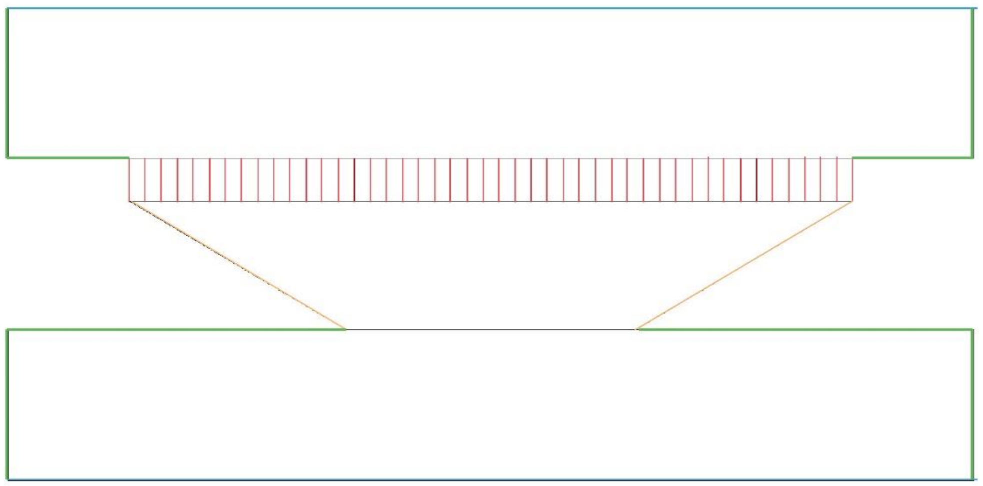

Fig. S25 Computer domain and boundaries of FEM models.

Table S2. Boundary conditions of FEM models.

Boundary	Poisson equation	Nerst-Planck equation
Blue boundaries	V_1=applied voltage; V_2=0 V	C_1=C_2=0.5 mM
Green boundaries	Zero charge/symmetry	Insulation/symmetry
Yellow boundaries	$\vec{n} \cdot \nabla\varphi = -\frac{\sigma_1}{\varepsilon}$	Insulation/symmetry
Red boundaries	$\vec{n} \cdot \nabla\varphi = -\frac{\sigma_2}{\varepsilon}$	Insulation/symmetry

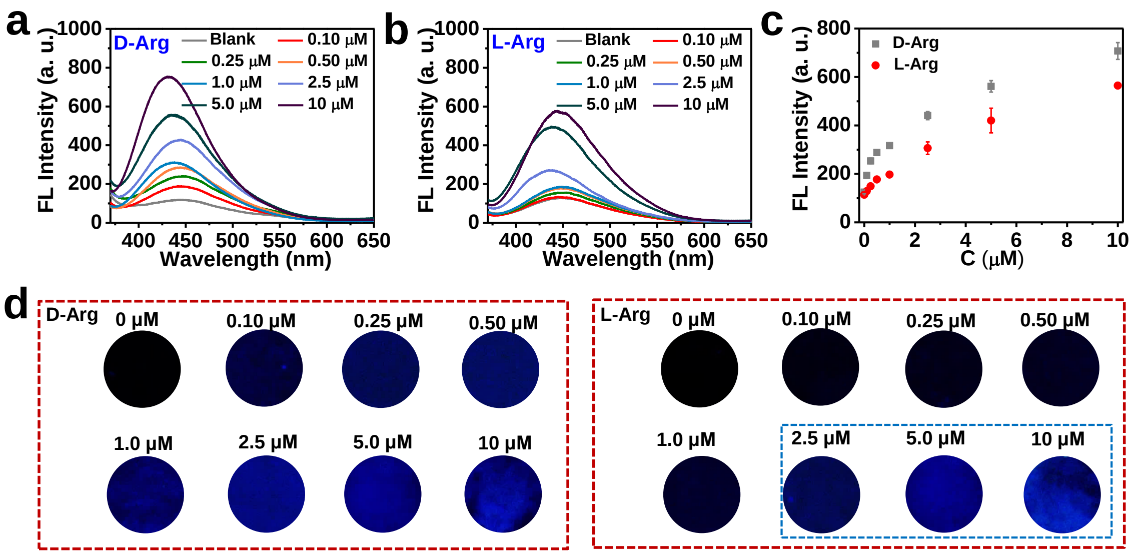

Fig. S26. Solid-state fluorescence spectrum for sensing different concentrations (0.1 µM–10 µM) of (a) D-Arg and (b) L-Arg. (c) Fluorescence intensity at 450 nm for sensing different concentrations of L- and D-Arg. (d) Fluorescence images of β-CD-CuMOF/TiO_2M for different L- or D-Arg concentrations (from 0.1 µM to 10 µM).

Reference

(S1) D. Momotenko and H. H. Girault, *J. Am. Chem. Soc.*, 2011, **133**, 14496−14499.

(S2) H. Daiguji, *Chem. Soc. Rev.*, 2010, **39**, 901−911.

(S3) Z. Y. Sun, F. Zhang, X. Y. Zhang, D. M. Tian, L. Jang and H. B. Li, *Chem. Commun.*, 2015, **51**, 4823–4826.

(S4) R. X. Cui, Y. Y. Wan, G. F. Ji and Z. L. Liu, *Analyst*, 2019, **144**, 5875–5881.

(S5) X. Q. Zeng, L. Zhang, J. D. Yang, Y. Guo, Y. M. Huang, H. Y. Yuan and Y. S. Xie, *New J. Chem.*, 2017, **41**, 15216–15228.

(S6) F. Temela, S. Erdemirc, E. Ozcelika, B. Tabakcic and M. Tabakci, *Talanta*, 2019, **204**, 172–181.
